# The Heart and Cannabis (THC) Cohort: Differences in Baseline Health and Behaviors by Cannabis Use

**DOI:** 10.1007/s11606-021-07302-6

**Published:** 2022-01-10

**Authors:** Salomeh Keyhani, Beth E. Cohen, Marzieh Vali, Katherine J. Hoggatt, Dawn M. Bravata, Peter C. Austin, Emily Lum, Deborah S. Hasin, Carl Grunfeld, Michael G. Shlipak

**Affiliations:** 1 Department of Medicine, University of California, San Francisco, San Francisco, CA USA; 2 San Francisco Veterans Administration, San Francisco, CA USA; 3 Northern California Institute for Research and Education, San Francisco, CA USA; 4 Medicine Service, Richard L. Roudebush VA Medical Center, Indianapolis, IN USA; 5 Department of Medicine, Indiana University School of Medicine, Indianapolis, IN USA; 6 University of Toronto, Toronto, Canada; 7 Mailman School of Public Health, Columbia University, New York, NY USA; 8 Department of Epidemiology and Biostatistics, University of California, San Francisco, San Francisco, CA USA

**Keywords:** cannabis, coronary artery disease, cohort, risk factors, smoking

## Abstract

**Background:**

Evidence on the cardiovascular health effects of cannabis use is limited. We designed a prospective cohort study of older Veterans (66 to 68 years) with coronary artery disease (CAD) to understand the cardiovascular consequences of cannabis use. We describe the cohort construction, baseline characteristics, and health behaviors that were associated with smoking cannabis.

**Objective:**

To understand the cardiovascular consequences of cannabis use.

**Design:**

We designed a prospective cohort study of older Veterans (66 to 68 years) with CAD.

**Participants:**

A total of 1,015 current cannabis smokers and 3,270 non-cannabis smokers with CAD.

**Main Measures:**

Using logistic regression, we examined the association of baseline variables with smoking cannabis in the past 30 days.

**Results:**

The current cannabis smokers and non-current smokers were predominantly male (97.2% vs 97.1%, *p*=0.96). Characteristics associated with recent cannabis use in multivariable analyses included lack of a high school education (odds ratio [OR] 2.15, 95% confidence interval [CI]: 1.10 to 4.19), financial difficulty (OR 1.47, 95% CI: 1.02 to 2.11), tobacco use (OR 3.02, 95% CI: 1.66 to 5.48), current drug use (OR 2.82, 95% CI: 1.06 to 7.46), and prior drug use (OR 2.84, 95% CI: 2.11 to 3.82). In contrast, compared to individuals with 0 to 1 comorbid conditions, those with 5 chronic conditions or more (OR 0.43, 95% CI: 0.27 to 0.70) were less likely to smoke cannabis.

**Conclusions:**

In this older high-risk cohort, smoking cannabis was associated with higher social and behavioral risk, but with fewer chronic health conditions.

**Supplementary Information:**

The online version contains supplementary material available at 10.1007/s11606-021-07302-6.

## INTRODUCTION

Cannabis is legal in 33 states and in Washington DC for medicinal purposes, and is now legal for recreational use in 15 states.^1^ This legalization has been accompanied by increased use of different forms of cannabis. Although the risks and benefits of cannabis have been inadequately studied, there is a general perception that it is safe and has health benefits.^2,3^

It is important to understand the impact of smoking cannabis on cardiovascular disease—the leading cause of morbidity and mortality in the USA.^4^ If cannabis has appreciable adverse cardiovascular effects, then it may contribute to this important public health burden. Several observations contribute to the hypothesis that cannabis use could be associated with poor cardiovascular outcomes. Endocannabinoid receptors are ubiquitous in the cardiovascular system. Cannabis use is associated with tachycardia,^5^ increased myocardial oxygen demand and platelet activation, as well as endothelial dysfunction and oxidative stress.^6–9^ Moreover, smoking cannabis (the predominant method of consumption)^10^ increases blood carboxyhemoglobin concentrations, and does so at a fivefold higher level than tobacco smoke.^11,12^ In studies that have compared tobacco smoke to cannabis smoke, cannabis appears to be more toxic than tobacco smoke in terms of particulate matter, toxins, and tar levels.^11,13,14^ Additionally, in rat models, cannabis smoke has a more prolonged impact on endothelial dysfunction (a key process leading to coronary disease) than tobacco smoke.^15^

Despite this strong evidence from physiologic and animal studies, few epidemiologic studies have evaluated the impact of cannabis on cardiovascular risk factors and events.^16^ Research has also been hindered by a lack of longitudinal studies of cannabis use among older cohorts, who have the highest risk for cardiovascular events. Existing cardiovascular cohorts have few cannabis users and low cumulative exposure of cannabis.^17,18^ Most studies have been conducted among young populations who are not at high risk for cardiovascular disease.^16^ To understand the potentially large and growing attributable risk from cannabis, the National Academies of Sciences, Engineering, and Medicine have called for cohort studies that could address the major gaps in research on the association of cannabis use with cardiovascular health.^19^ This will require rapid establishment of cohorts that are both large in size and comprehensive enough to examine the likely confounders of hypothesized relationships between cannabis use and cardiovascular health, e.g., tobacco use, substance use, adherence to medications (e.g., statins), and other health behaviors that may accompany cannabis use.^20^

In 2018, we launched a prospective cohort study designed to examine the cardiovascular effects of cannabis use in an older Veteran population. We focused on older patients with existing cardiovascular disease to address the challenges of prior studies (e.g., low event rates). In this paper, we describe the novel approach employed in cohort construction and the baseline characteristics of the THC cohort. We also examine factors associated with smoking cannabis in this older cohort. This information will be informative for the development of other prospective cohorts and for the identification of baseline factors that will be critical to account for when analyzing the association of cannabis use and cardiovascular outcomes.

## METHODS

### Cohort Construction

THC cohort construction involved several steps: (1) identifying community dwelling Veterans 66 to 68 years of age; (2) preliminarily categorizing patients using text processing methods into cannabis users and non-users;(3) interviewing a random sample of potential cannabis users and non-users (based on text processing tool) using a health interview tool with validated items designed to capture cannabis use and other important baseline characteristics; (4) merging the health interview data with national VA data to create a cohort with detailed data on demographic characteristics, socioeconomic factors, health behaviors including physical activity and clinical conditions. Details of each step provided below:

### Sampling Strategy/Cohort Design

We constructed a cohort of patients 66 to 68 years of age with coronary artery disease (CAD) in the Veterans Health Administration (VA) in 2018 who were cared for in VA primary care. The study was designed to focus on older Veterans with CAD because the secondary prevention setting offers smaller sample size requirements. In addition, patients with prevalent CAD should be receiving guideline concordant medical treatment so we can evaluate the association of cannabis use with the achievement of secondary prevention goals. We chose Veterans who were at least age 66 to ensure that at least 1 year of baseline Medicare data was available for comprehensive characterization of the cohort and complete assessment of outcomes. We chose a narrow age range to reduce the influence of age-related variability in the risk for outcomes.

**Figure 1.**
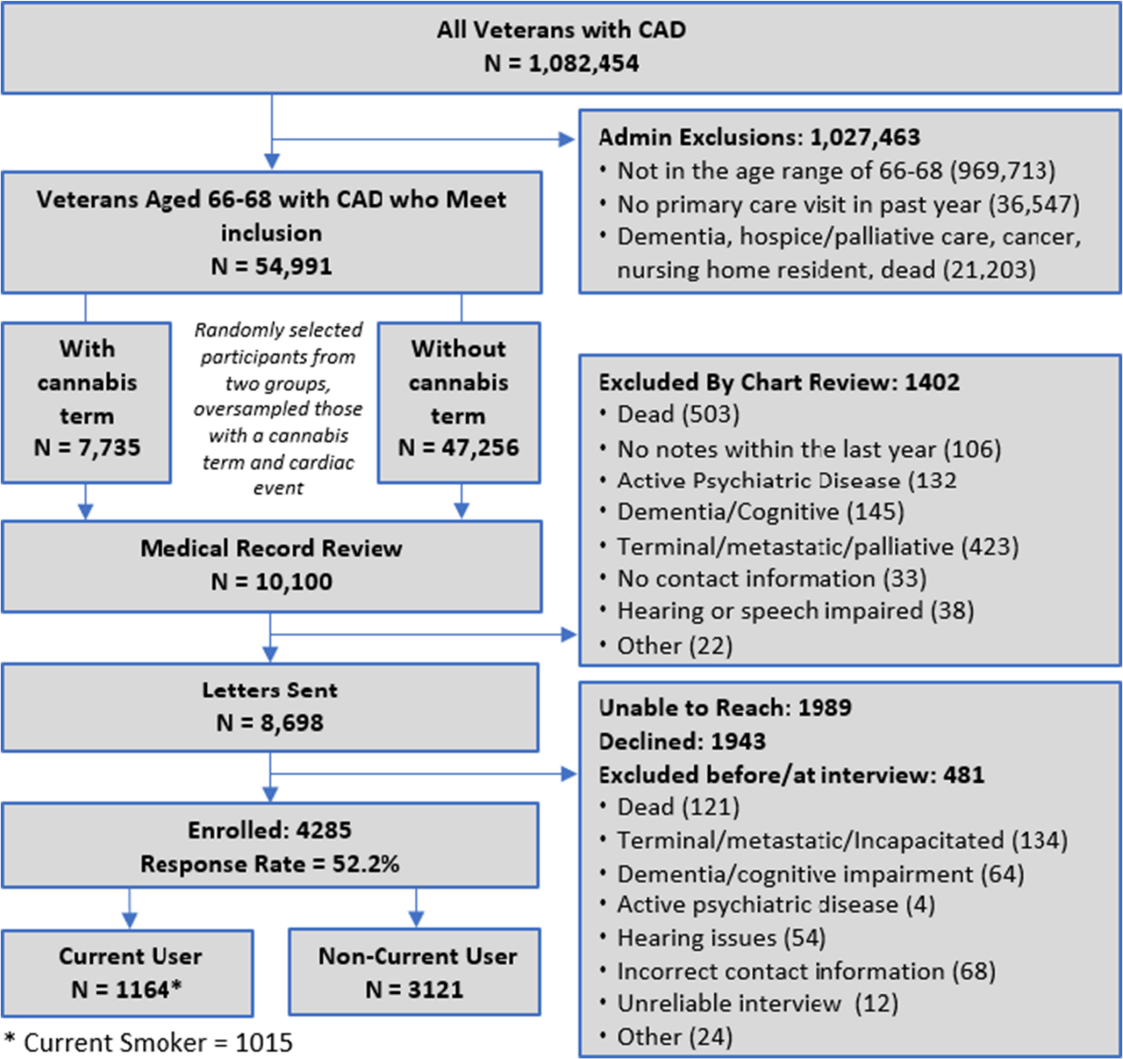
Cohort construction

We identified all Veterans with an inpatient or outpatient International Classification of Diseases (ICD) code for CAD (Supplement 1) in the past 5 years in the VA (*N*=1,082,454) (Figure 1). We excluded those without a VA primary care visit in the last year to ensure that we captured a population who would have baseline data available in the VA. We used administrative data to exclude potential participants who had dementia, who were receiving end of life care, who were residing in nursing homes, and who were receiving active cancer treatment. These exclusions left 54,991 community dwelling patients with CAD eligible for participation in the study.

To identify cannabis users among the 54,991 patients, as a first step, we used a previously developed text processing algorithm^19^ to categorize potential participants into two groups: those with a term denoting cannabis use in their medical record in the past 6 months (*n*=7,735) (e.g., cannabis, marijuana, MJ) and those without a cannabis term (47,256) in the past 6 months. Our previous work suggested that the presence of a term denoted current or former use and rarely denoted “negation” or non-use.^19^ We developed a lexicon describing cannabis use (Supplement 2). Our goal was to recruit at least 800 current cannabis smokers, defined as use in the past 30 days. We randomly selected individuals from both groups to recruit a mix of those initially identified as potential cannabis smokers and non-smokers in batches of 25 to 50 patients. Among this sample of individuals with CAD, we also oversampled participants who had experienced a cardiovascular event (AMI, stroke, or revascularization) using ICD codes (Supplement 1) in the prior 5 years from both groups to enhance our recruitment of a high-risk cohort. Once participants had been selected, we reviewed medical charts to identify those with exclusions missed by administrative data. We also excluded individuals who were unable to consent and participate in a telephone interview, including those with evidence of active psychosis, cognitive impairment, and speech and hearing deficits. Among 10,100 Veterans sampled from the VA population, 1,402 were excluded at the medical record review phase, and 8,698 were sent letters of invitation to participate. Among the 8,698 who were sent letters, we were unable to reach 1,989 participants, 1,943 declined, and 481 were excluded after the letter was sent because they met an exclusion criterion that was identified before or during the interview. We recruited 4,285 participants (recruitment rate 4,285/8,217, 52%) from April 5, 2018 through March 12, 2020, of whom 1,164 used a form of cannabis in the past 30 days. Among those 1,164 cannabis users, 1,015 reported smoking cannabis in the prior 30 days in a telephone interview (current cannabis smokers) (Figure 1). This cohort study was approved by the University of California, San Francisco Institutional Review Board.

### Data Collection

We obtained study data from the VA Corporate Data Warehouse (CDW), Medicare utilization files, and telephone interviews.^21^ Telephone interviews were approximately 20 min long, and participants were provided a $20 incentive. Interviewers received training prior to starting data collection (Supplement 3), and calls were recorded for quality assurance purposes. Every week a random sample of interviews were reviewed by the study team project manager. Interviewers obtained verbal informed consent from participants over the telephone, as approved by the University of California, San Francisco Institutional Review Board.

#### Assessment of Cannabis Use in a Telephone Interview

We previously developed and tested a tool to assess the forms, frequency, and duration of cannabis use in a sample of 339 Veterans with CAD.^22^ We assessed forms of cannabis used in the past 30 days, including smoking, vaping, dabbing, and edibles. Our main exposure assessment in the current study was smoking cannabis in the past 30 days (current use) and was asked with the following question: “Have you smoked marijuana in the past 30 days?” This question categorized cannabis users as either current cannabis smokers or non-current smokers in the past 30 days. We also assessed forms of smoking (use of joints, pipes, and bongs) and asked participants the number of days per week they used cannabis in the past month for each form of smoking. We queried participants about frequency of use on days used with the question: “On the days you smoked in the last 30 days, how many joints/pipes/bongs did you smoke a day?” This allowed assessment of total frequency of use in the prior 30 days. We also assessed combined use of tobacco and cannabis (blunts, spliffs). Finally, we assessed lifetime use with the question “Over the entire period you smoked marijuana, how many years did you smoke marijuana on a daily or near-daily basis?”.

#### Assessment of Baseline Health Using the Telephone Interview

We designed the interview instrument to be easy to understand over the telephone and to assess domains of health with a strong and established relationship with cardiovascular events. We used validated survey items that have been successfully implemented in previous studies (Supplement 4), and that covered domains for tobacco exposure history, alcohol and drug use, mobility, physical activity, depression, and post-traumatic stress disorder (PTSD). We measured tobacco use with questions adapted from the Psychiatric Research Interview for Substance and Mental Disorders for DSM-V^23^ and the National Health Interview Survey.^24^ We assessed high-risk alcohol use with the 3-item Alcohol Use Disorders Identification Test – Concise^25^ and illicit drug use with questions adapted from Coronary Artery Risk Development in Young Adults.^26,27^ We collected mobility information and physical activity using questions from the Health and Retirement Study^28^ and the International Physical Activity Questionnaire,^29^ respectively. We measured depressive symptoms with the Patient Health Questionnaire-9^30,31^ and PTSD with the PTSD Checklist-5.^32^ We collected data on self-reported health using the Short Form Survey,^33^ and questions on socioeconomic status (marital status, education, housing, number of individuals in household, poverty) with questions adapted from Coronary Artery Risk Development in Young Adults and the Health and Retirement Study.^34^

#### Assessment of Baseline Health Using VA Data and Medicare Data

We used VA CDW^35^ data for our measures of demographics (age, sex, and race/ethnicity) and cardiovascular risk factors (hypertension, hyperlipidemia, and diabetes using ICD9/ICD10 codes from both VA and Medicare data). We used vital signs data for blood pressure (measured in primary care visits) and height and weight (from which we computed body mass index). We also defined measures for the presence of chronic conditions (peripheral vascular disease, congestive heart failure, atrial fibrillation, chronic kidney disease, chronic obstructive lung disease, pulmonary fibrosis, and sleep apnea). For each clinical condition, we deemed the condition present if the participant had at least two outpatient visits or one inpatient visit with the ICD10 diagnosis code in the past 2 years. We also created a variable representing each participant’s total number of comorbid conditions (0–1, 2, 3, 4, 5+) using the previously outlined conditions. We estimated a *CHA*_*2*_*DS*_*2*_*-VASc* Score, a Charlson Comorbidity Index, and extracted the VA Care Assessment Need (CAN score) closest to the interview data. Finally, we extracted laboratory data from the VA CDW for kidney function and lipid profiles of the participants.

### Statistical Analysis

We computed participant-specific weights to account for the oversampling of patients who had a cardiovascular event prior to the index interview. These weights were used so that the weighted sample would be representative of the VA population ages 66–68 with CAD. Using these weights, we computed descriptive statistics for each of the baseline characteristics of the cohort. We then examined the distribution of cannabis use in the cohort. We estimated the frequency of cannabis use by querying each form of smoking (joint, pipe, bong) in the past 30 days and asking the number of times used each day of reported use. We also categorized use by different forms used in the past 30 days. We first compared each baseline characteristic’s association with current cannabis use in an unadjusted logistic regression model. To estimate the association between baseline variables and cannabis smoking in the past 30 days, we fit logistic regression models that incorporated the study weights. We included variables in the model from all domains hypothesized to be associated with cannabis use including sociodemographic factors, health behaviors, self-reported health, mobility, mental health, and number of comorbid conditions. R Statistical software (R-4.03) was used for the analyses.

### Patient and Public Involvement

No members of the public were involved in the design, conduct, reporting, or dissemination of the research. Results will be disseminated to participants through https://phprg.ucsf.edu after completion of the main study.

## RESULTS

The recruitment rate was 52% (58% recreational, 50% medically legal, and 52% non-legal states). Users were recruited from 49 States and the District of Columbia. South Dakota was the only state from which a user was not recruited. The top 10 states from which participants were recruited included the following: California (178, 15.3%), Michigan (94, 8.1%), Florida (76, 6.5%), Texas (52, 4.5%), Arizona (51, 4.4%), Ohio (50, 4.3%), New York (48, 4.1%), Oregon (40, 3.4%), Wisconsin (39, 3.4%), and Colorado (33, 2.8%).

The cohort includes 1,164 cannabis users and 3,121 non-current users. Among the 1,164 cannabis users, 1,015 reported smoking cannabis (focus of this analysis), and 3,270 did not smoke cannabis. The weighted prevalence of cannabis use in this population in the past 30 days was 11%. Table 1 shows the baseline characteristics of the cohort. There were differences in baseline socioeconomic factors between current smokers and non-current smokers. Current smokers were less likely to be married (41.9% vs. 56%) or employed (10.3% vs. 13.1%). Current smokers more commonly lived alone (39.3% vs. 29.2%) and more commonly reported financial difficulty (paying for basics was hard or very hard) (21.1% vs. 13.6%). Cannabis smokers had a higher prevalence of current tobacco use (43.9% vs. 23%), exposure to secondhand smoke (35.7% vs. 12.3%), and high-risk drinking (33% vs. 18.4%). Although current use of illicit drugs was infrequent in this older cohort, past illicit drug use was more common among cannabis smokers (53.5% vs. 20.6%). In contrast, current smokers had a lower prevalence of chronic health conditions compared to non-smokers, including hypertension (80.9% vs. 83.6%), diabetes (37.8% vs. 50.3%), and obesity (mean body mass index 29.7 vs. 32.4).

**Table 1.** The Heart and Cannabis(THC) Cohort: Baseline Characteristics of Veterans with Coronary Artery Disease Who Reported Current Cannabis Smoking and Non-smokers^a^

	Current cannabis smoker (*N* = 1015)	Non-current smoker (*N* = 3270)	*p*-value
Age (mean)	67.8	68.1	<0.001
Male	996 (97.2%)	3200 (97.1%)	0.96
Race			0.31
White	794 (81.9%)	2611 (83.6%)	
Black	193 (15.2%)	521 (12.5%)	
Other	28 (2.9%)	138 (4%)	
Hispanic or Latino	56 (7.5%)	137 (3.5%)	0.001
Married	408 (41.9%)	1750 (56%)	<0.0001
Education			0.0003
Less than high school graduate	103 (8.6%)	223 (5.8%)	
High school/some college degree	794 (80.8%)	2479 (75.6%)	
Bachelors and beyond	117 (10.6%)	550 (18.1%)	
Unknown	1 (0.1%)	18 (0.6%)	
Stable housing situation	965 (96.9%)	3155 (97.3%)	0.59
Lives alone	372 (39.3%)	976 (29.2%)	<0.001
Paying for basics very hard or hard	236 (21.1%)	521 (13.6%)	<0.001
Employed	78 (10.3%)	365 (13.1%)	0.23
Physical Activity (IPAQ)			0.039
Low	386 (34.8%)	1378 (40%)	
Moderate	282 (27.1%)	898 (29.3%)	
High	347 (38.1%)	994 (30.7%)	
Audit score (>=4 for men, >=3 for women)	316 (33%)	616 (18.4%)	<0.0001
Tobacco smoking			<0.0001
Current	465 (43.9%)	826 (23%)	
Former	500 (52.2%)	2074 (64.5%)	
Never	50 (3.8%)	370 (12.5%)	
Secondhand smoke exposure	423 (35.7%)	459 (12.3%)	<0.0001
Current illicit drug use	35 (2.8%)	23 (0.4%)	<0.0001
Past other illicit drug use	597 (53.5%)	949 (20.6%)	<0.0001
Use of other forms of cannabis	266 (19.4%)	149 (2.6%)	<0.0001
Self-reporting health			0.38
Excellent/Very Good	36 (3.5%)	142 (5.4%)	
Good	406 (45.1%)	1240 (42.7%)	
Fair/Poor	572 (51.4%)	1887 (51.8%)	
Refused/Don't know	1 (0%)	1 (0.1%)	
Health compared to last year			0.30
Much better or Somewhat better	267 (27.3%)	847 (23.3%)	
About the same	522 (51.1%)	1648 (54.6%)	
Somewhat worse or Much worse	226 (21.5%)	775 (22.1%)	
Mobility			0.31
None	260 (25.2%)	800 (23.7%)	
1 block	353 (31%)	1156 (30.9%)	
Several blocks	150 (17%)	553 (21.5%)	
1 mile	251 (26.9%)	759 (23.8%)	
Depressed (PHQ>9)	356 (28.2%)	1151 (27.6%)	0.84
PTSD (PHQ > 18)	135 (11%)	498 (10.5%)	0.77
Cardiovascular risk factors			
Hypertension	822 (80.9%)	2788 (83.6%)	0.28
Hyperlipidemia	699 (72%)	2568 (78.4%)	0.014
Diabetes	365 (37.8%)	1596 (50.3%)	<0.001
BMI (mean)	29.7	32.4	<0.0001
Systolic Blood Pressure (mean)	132.26	131.39	0.28
Diastolic Blood Pressure (mean)	75.39	74.65	0.13
Total Cholesterol (mean)	159.57	151.49	0.003
HDL (mean)	44.91	41.79	0.001
LDL (mean)	88.62	82.82	0.006
Obesity	422 (43.1%)	1916 (59.1%)	<0.0001
Cardiovascular events in past 5 years			
Percutaneous Coronary Intervention	152 (13.8%)	666 (20.4%)	0.0032
Coronary artery bypass graft	72 (5.8%)	243 (6.4%)	0.67
Lower extremity revascularization	49 (3.8%)	109 (2.9%)	0.36
Acute myocardial infarction	163 (13.7%)	616 (18.1%)	0.033
Stroke	33 (2.3%)	181 (5.5%)	0.0022
Other conditions			
Transient ischemic attack	29 (2.5%)	74 (1.9%)	0.40
Peripheral vascular disease	78 (7.1%)	267 (7.7%)	0.69
Congestive heart failure	176 (15.6%)	770 (20.5%)	0.034
Atrial fibrillation	137 (11.1%)	593 (16.2%)	0.011
Other cardiac arrhythmia	115 (10.7%)	516 (13.6%)	0.17
Abdominal aortic aneurysm	56 (5.1%)	151 (3.8%)	0.30
Defibrillator	15 (0.7%)	58 (1.6%)	0.0055
Chronic kidney disease			
GFR 30 to 60	168 (14%)	675 (17.7%)	0.083
GFR<30	29 (2.1%)	113 (2.7%)	0.49
Prostate cancer	45 (4%)	177 (4.8%)	0.50
Chronic obstructive pulmonary disease	309 (28%)	928 (24.1%)	0.15
Pulmonary fibrosis	12 (1.3%)	43 (0.7%)	0.29
Sleep apnea	229 (22.5%)	1153 (32.7%)	<0.001
Pneumonia	75 (5.8%)	289 (6.2%)	0.80
Deep vein thrombosis/pulmonary embolism	28 (2.3%)	105 (2.6%)	0.77
Rheumatoid arthritis	21 (2.1%)	60 (1.6%)	0.48
Charlson score	3.9 (1.78)	4.3 (1.91)	<0.001
CAN score			0.039
≤25	16 (0.8%)	81 (2.9%)	
25–50	260 (28.3%)	904 (31.8%)	
50–75	453 (45.7%)	1359 (41.1%)	
≥75	286 (25.2%)	926 (24.1%)	
CHA_2_DS_2_-VASc score	2.7 (1.21)	3 (1.29)	<0.001
Number of comorbid conditions^b^			<0.001
0–1	184 (17.5%)	339 (11.4%)	
2	227 (23.9%)	542 (18%)	
3	231 (25.1%)	760 (25.5%)	
4	181 (16%)	691 (21.1%)	
5+	192 (17.5%)	938 (24%)	

*Audit*, Alcohol Use Disorders Identification Test; *BMI*, body mass index; *GFR*, glomerular filtration rate; *HDL*, high-density lipoprotein; *IPAQ*, International Physical Activity Questionnaire; *LDL*, low-density lipoprotein; *PCL-5*, PTSD Checklist for DSM-5;*PHQ-9*, Patient Health Questionnaire-9

^a^Weighted to account for complex sampling design, percentages are weighted percents

^b^Hypertension, hyperlipidemia, obesity (BMI >30), peripheral vascular disease, congestive heart failure, atrial fibrillation, chronic kidney disease, chronic obstructive pulmonary disease, sleep apnea, pulmonary fibrosis

### Patterns of Cannabis Use

**Figure 2.**
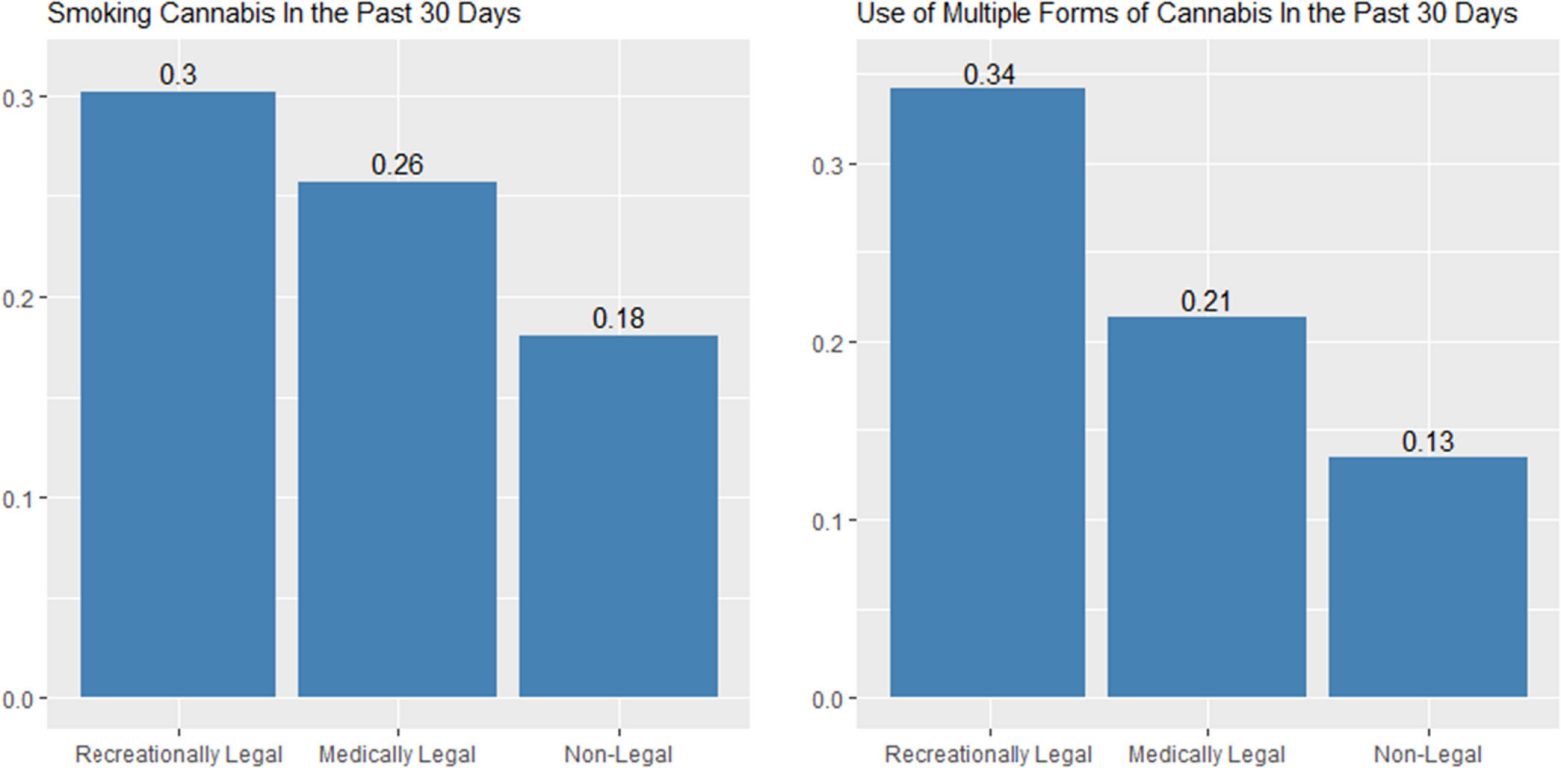
Use patterns across recreationally and medically legal and non-legal states.

Among the 1,015 current smokers, 510 (43.3%) used cannabis daily (Table 2). About 28% reported smoking in more than one form (e.g., joint, pipe, bong, spliff, blunt) in the past 30 days. Current smokers also frequently used other forms of cannabis, with 9.2% reporting vaping and 9.7% reporting edible use, but few reporting dabbing (1.6%). About 30% of current smokers reported that cannabis use was for medical reasons, and pain was the most reported reason for use. Supplement 6 displays the distribution of frequency of use among the current cannabis smokers. Current smokers, on average, reported smoking 76 times per month (median 30, interquartile range [IQR] 4 to 120). Among 510 participants who reported daily use, the average number of times used per day was 3.9 (median 3, IQR 1 to 5). Figure 2 demonstrates that most participants resided in recreationally legal states (30%), followed by medical states (26%) and non-legal states (18%). Use of multiple forms of cannabis was more common in recreationally legal states (Figure 2).

**Table 2.** Cannabis Use Patterns Among Current Cannabis Smokers (Used in the Past 30 Days) ^a^, *N*=1,015

	Current smokers (joint/pipe/bong)
Frequency of use in past 30 days	*N* (weighted)%
Daily smoking	510 (43.3%)
Near daily smoking (>20 days of use)	573 (48%)
Number of times used in past 30 days (mean, median, interquartile range)	(76.2, 30, 4-120)
Number of times daily users used per day (mean, median, interquartile range)	(3.9, 3, 1-5)
Forms of smoking in past 30 days	*N* (weighted)%
Joints	653 (65.2%)
Pipes	531 (51.7%)
Bongs	76 (6.8%)
Blunts	90 (7.7%)
Spliffs	23 (1.5%)
Smoked in more than one form in past 30 days	303 (28%)
Forms of cannabis use in past 30 days	
Vaping in past 30 days	124 (9.2%)
Edible use in past 30 days	125 (9.7%)
Dabbing in past 30 days	26 (1.6%)
Topical use in past 30 days	73 (4.8%)
Type of use	
Medically	310 (30.1%)
Recreationally	101 (10.7%)
Both	600 (58.6%)
Mean years of daily or near daily use (se)	18.8 (16.24)
Reasons for use	
Pain	508 (49.5%)
Post-traumatic stress disorder	116 (10.3%)
Sleep	53 (5.2%)
Other reason	338 (35%)

^a^Weighted to account for complex sampling design, weighted percentages presented

### Association of Baseline Health Characteristics with Smoking Cannabis

In multivariable analyses adjusting for baseline characteristics, Hispanic ethnicity was associated with greater odds of current cannabis smoking (odds ratio [OR] 2.92, 95% confidence interval [CI]: 1.51 to 5.66) relative to white race, but we observed little evidence of an association for black race (OR 1.06, 95% CI: 0.73 to 1.53) relative to white race (Table 3). Those without a high school education (OR 2.15, 95% CI: 1.10 to 4.19) were more likely to smoke cannabis compared to those with bachelor’s degrees. Cannabis smoking was also more common among individuals who reported financial difficulty (OR 1.47, 95% CI: 1.02 to 2.11), and was less common among employed (OR 0.71, 95% CI: 0.43 to 1.18) individuals. Current cannabis smoking was less common among individuals reporting low levels of physical activity (OR 0.62, 95% CI: 0.43 to 0.88) and more common among those reporting tobacco smoking (current tobacco use: (OR 3.02, 95% CI: 1.66 to 5.48); former tobacco use: (OR 2.08, 95% CI: 1.20 to 3.62)). Current cannabis smoking was also more common among individuals exposed to secondhand smoke (OR 2.51, 95% CI: 1.82 to 3.47) or who engaged in current (OR 2.82, 95% CI: 1.06 to 7.46) or past illicit drug use (OR 2.84, 95% CI: 2.11 to 3.82). Current cannabis smokers were also more likely to use other forms of cannabis (OR 7.91, 95% CI: 5.13 to 12.2). There were no differences in PTSD among current cannabis smokers compared to non-smokers. Compared to individuals with 0–1 comorbid conditions, those with 5 chronic conditions (OR 0.43, 95% CI: 0.27 to 0.70) were less likely to smoke cannabis.

**Table 3.** Multivariable Analysis Comparing Baseline Sociodemographic, Mental Health, and Behavioral Risk Factors Associated with Smoking Cannabis in Past 30 Days^a^

	Unadjusted OR (95%CI)	Adjusted OR (95%CI)
Age (mean)	0.82 [0.74, 0.91]	0.88 [0.78, 0.99]
Male	1.03 [0.40, 2.63]	0.90 [0.37, 2.17]
Race		
White (reference)		
Black	1.27 [0.92, 1.76]	1.06 [0.73, 1.53]
Other	0.71 [0.42, 1.23]	0.58 [0.28, 1.19]
Hispanic or Latino	2.28 [1.24, 4.19]	2.92 [1.51, 5.66]
Married	0.56 [0.44, 0.73]	0.80 [0.57, 1.12]
Education		
Less than high school graduate	2.53 [1.51, 4.24]	2.15 [1.10, 4.19]
High school/some college degree	1.83 [1.26, 2.65]	1.47 [0.92, 2.34]
Bachelors and beyond (reference)		
Stable housing situation	0.84 [0.45, 1.56]	1.30 [0.58, 2.92]
Lives alone	1.57 [1.21, 2.04]	1.12 [0.79, 1.61]
Paying for basics very hard or hard	1.70 [1.27, 2.27]	1.47 [1.02, 2.11]
Employed	0.76 [0.48, 1.19]	0.71 [0.43, 1.18]
Physical activity (IPAQ)		
Low	0.70 [0.52, 0.93]	0.62 [0.43, 0.88]
Moderate	0.74 [0.54, 1.03]	0.62 [0.43, 0.9]
High (reference)		
Audit score (≥4 for men, ≥3 for women)	2.18 [1.65, 2.89]	1.83 [1.33, 2.52]
Tobacco smoking		
Current	6.23 [3.76, 10.34]	3.02 [1.66, 5.48]
Former	2.64 [1.61, 4.34]	2.08 [1.2, 3.62]
Never (reference)		
Secondhand smoke exposure	3.94 [2.99, 5.19]	2.51 [1.82, 3.47]
Current drug use	7.75 [2.91, 20.65]	2.82 [1.06, 7.46]
Past other drug use	4.44 [3.44, 5.74]	2.84 [2.11, 3.82]
Use of other forms of cannabis	8.9 [6.11, 12.98]	7.91 [5.13, 12.2]
Self-reporting health		
Excellent/very good (reference)		
Good	1.65 [0.86, 3.18]	1.50 [0.74, 3.04]
Fair/poor	1.55 [0.81, 2.96]	1.26 [0.61, 2.58]
Health compared to last year		
Much better/somewhat better (reference)		
About the same	0.80 [0.59, 1.07]	0.74 [0.53, 1.04]
Much worse/somewhat worse	0.83 [0.58, 1.18]	0.76 [0.49, 1.16]
Mobility		
None/1 block	0.91 [0.67, 1.24]	1.06 [0.74, 1.53]
Several blocks	0.70 [0.47, 1.04]	0.77 [0.50, 1.2]
1 mile (reference)		
Depressed (PHQ>9)	1.03 [0.80, 1.32]	1.04 [0.74, 1.45]
PTSD (PHQ > 18)	1.05 [0.74, 1.5]	0.86 [0.55, 1.36]
Number of comorbid conditions^b^		
0–1 (reference)		
2	0.86 [0.56, 1.31]	0.81 [0.51, 1.28]
3	0.64 [0.43, 0.96]	0.59 [0.37, 0.93]
4	0.49 [0.32, 0.75]	0.43 [0.27, 0.68]
5+	0.47 [0.31, 0.72]	0.43 [0.27, 0.70]

*Audit*, Alcohol Use Disorders Identification Test; *IPAQ*, International Physical Activity Questionnaire; *PCL-5*, PTSD Checklist for DSM-5;*PHQ-9*, Patient Health Questionnaire-9

^a^The estimates include personal level weights to account for sampling design

^b^Hypertension, hyperlipidemia, obesity (BMI >30), peripheral vascular disease, congestive heart failure, atrial fibrillation, chronic kidney disease, chronic obstructive pulmonary disease, sleep apnea, pulmonary fibrosis

## DISCUSSION

In this report on the baseline characteristics of the THC cohort, we found that smoking cannabis was associated with social risk factors and adverse health behaviors. We also found that smoking cannabis was less common among those with a greater number of chronic health conditions. The development of the THC cohort also demonstrates it is feasible to efficiently recruit a cohort of current cannabis users and non-current users using data on cannabis use extracted from the free text of the electronic health record.

Our finding that cannabis use is associated with social risk factors and adverse health behaviors has been demonstrated in the prior literature but primarily among younger adults. Previous research on cannabis use among younger adults found that those who used cannabis were more frequently unemployed, unmarried, with lower educational status, and more likely to use other substances.^36–38^ Other studies have also demonstrated that co-use of tobacco and cannabis is common although the evidence is strongest in younger adults.^36^ There are little data on factors associated with cannabis use among older adults. One study found that older adults who used cannabis were less likely to be married and more likely to reside in recreational states, but the study did not include information on health behaviors and comorbid conditions.^39^ This study suggests that cannabis use among older adults is also associated with other adverse health behaviors (e.g., tobacco use, alcohol use, and drug use).

We also found that individuals who smoked cannabis had fewer comorbid conditions and engaged in more physical activity. Therefore, there may be a relationship between health status and use or a relationship between quitting cannabis use and health, similar to what has been reported among tobacco users. Recent quitters of tobacco may have a health reason to quit so non-current users may have a higher prevalence of comorbidity.^40,41^ A similar relationship has been reported among alcohol users where individuals who have quit are different from low-risk users.^42^ In other words, it is possible that individuals with multiple comorbidities or acute events may quit cannabis, resulting in the observed differences in baseline comorbidity between current users and non-current users.

The findings from this cohort have important implications for the study of the cardiovascular health effects of cannabis among older adults. Baseline data from the THC cohort suggest that studies of the effects of cannabis on health need to accurately assess the presence of key baseline behavioral health factors and clinical factors to account for other factors associated with cardiovascular events. It is possible that some studies that report a health benefit from cannabis use may be in fact confounded by the fact that cannabis users are healthier. A detailed assessment of health status data is important in studies examining the health effects of cannabis use.

The detail on cannabis use collected in this cohort also suggests that tools that capture cannabis use need to account for the different forms of smoking cannabis and the different forms of cannabis available. About a third of those who smoked cannabis engaged in multiple modalities of smoking. In addition, they were more likely to use other forms of cannabis, with 9% reporting they also vaped and 9% reporting they also used edibles. Capturing all modalities of use may be particularly important for some outcomes (e.g., blood pressure) given the hemodynamic effects of cannabis.^5^ In addition, while the main exposure variable proposed for this cohort is use in the past 30 days, our data suggest that use patterns vary significantly among current users. Illustrating the importance of assessing frequency of current use, some participants reported using less than 10 times per month (e.g., weekend user) while about half reported using daily with an average frequency of almost 4 times per day. Patients with more intensive daily use may potentially be at even greater risk of adverse outcomes and could be an important group to recruit in studies of the cardiovascular effects of cannabis use.

Study limitations are noted. This first report of the THC is cross-sectional analysis and causality cannot be inferred. The THC cohort includes very few women as older Veterans are predominantly men. We recruited a high-risk cohort of elderly Veterans with CAD for the purpose of examining the association of current cannabis use with cardiovascular events. However, given the specific nature of the cohort, our findings may not generalize to other populations (e.g., non-Veterans, younger or very-old persons, cohorts without vascular disease and women). Our sample was also limited by the requirement for a telephone interview. Individuals with cognitive impairment, hearing, or speech deficits or those in nursing homes and/or in palliative care and hospice were excluded. Therefore, our cohort is more reflective of an ambulatory population of older adults and does not generalize to institutionalized individuals, those with severe end of life illness or individuals with cognitive impairment. In addition, given the over-sampling of individuals who smoked cannabis, the sample of users in the cohort cannot be interpreted as measures of the prevalence of cannabis use in the older VA population. Our cohort includes few individuals that vape cannabis. Despite these limitations, the substantial proportion of cohort members with current cannabis use, as well as the relatively high frequency of use should enable us to determine whether there are independent associations of cannabis use with longitudinal risk of adverse cardiovascular outcomes.

In conclusion, in this cross-sectional analysis of the THC cohort, smoking cannabis was associated with social risk factors (e.g., lack of high school degree, financial difficulty) and behavioral risk including current tobacco use, risky alcohol use, current and past drug use. In addition, current cannabis users had fewer comorbidities. Prospective studies examining the health effects of smoking cannabis will require detailed data on social risk, health behaviors, and health status as these factors are associated with both cannabis use and adverse health outcomes.

## Supplementary Information

ESM 1 (DOCX 40 kb)

## Data Availability

Data are available upon reasonable request. Access to the data can be permitted in accordance with Veterans Health Administration Policy.
