# Supplementary material for: The Heart and Cannabis (THC) Cohort: Differences in Baseline Health and Behaviors by Cannabis Use
Source: J Gen Intern Med. 2022 Jan 10;37(14):3535–44. doi: 10.1007/s11606-021-07302-6 (PMC9585109; doi:10.1007/s11606-021-07302-6)
Supplement: Supplementary file 1 — (DOCX 40 kb) [file 11606_2021_7302_MOESM1_ESM.docx]

Supplement 1. ICD9 and 10 Codes and CPT Codes

| **Condition** | **ICD9**  **Diagnosis codes** | **ICD10**  **Diagnosis codes** | **ICD9**  **Procedure codes** | **ICD10 Procedure codes** | **CPT codes** |
| --- | --- | --- | --- | --- | --- |
| **Coronary Artery Disease** | 410.xx, 411.xx, 412.xx, 414.xx, 429.7x | I21.xx, I22.xx, I23.xx, I24.xx, I25.xx | 00.66, 36.10, 36.11, 36.12, 36.13, 36.14, 36.15, 36.16, 36.17, 36.06, 36.07, 36.09, 36.19,  36.2 | 02109x, 02100Ax, 02100Jx, 02100Kx, 02100Zx, 021049x, 02104Ax, 02104Jx, 02104Kx, 02104Zx, 021109x, 02110Ax, 02110Jx, 02110Kx, 02110Zx, 021149x, 02114Ax, 02114Jx, 02114Kx, 02114Zx, 021209x, 02120Ax, 02120Jx, 02120Kx, 02120Zx, 021249x, 02124Ax, 02124Jx, 02124Kx, 02124Zx  021309x, 02130Ax, 02130Jx, 02130Kx, 02130Zx, 021349x, 02134Ax, 02134Jx, 02134Kx, 02134Zx, 027034x, 02703Dx, 02703Tx, 02703Zx, 027044x, 02704Dx, 02704Tx, 02704Zx, 027134x, 02713Dx, 02713T6, 02713ZZ, 02714ZZ, 02723ZZ, 02724ZZ, 02733ZZ, 02734ZZ | 33510, 33511, 33512, 33513, 33514, 33515, 33516, 33517, 33518, 33519, 33521, 33522, 33523, 33533, 33534, 33535, 33536, 33545, 33572, 4110F, 92973, 92974, 92980, 92981, 92982, 92984, 92995, 92996, 93540, 93564, G0290, G0291, S2205, S2206, S2207, S2208, S2209 |
| **Acute Myocardial Infarction** | 410.xx, 429.7xx | I21.xx, I22.xx, I23.xx |  |  |  |
| **Percutaneous Coronary Intervention** |  |  | 0.66, 36.03, 36.04, 36.06, 36.07, 36.09 | 0270xxx, 02C03Zx, 02C04Zx, 02C13Zx, 02C14Zx, 02C23Zx, 02C24Zx, 02C33Zx, 02C34Zx | 92920, 92921, 92924, 92925, 92928, 92929, C9600, C9601, 92933, 92934, C9602, C9603, 92937, 92938, C9604, C9605, 92943, 92944, C9607, C9608 |
| **Coronary Artery Bypass Graft** |  |  | 36.10, 36.11, 36.12, 36.13, 36.14, 36.15, 36.16, 36.17, 36.19 | 0210xxx, 0211xxx, 0212xxx, 0213xxx | 33510, 33511, 33512, 33513, 33514, 33516, 33517, 33518, 33519, 33521, 33522, 33523, 33533, 33534, 33535, 33536 |
| **Stroke** | 430.xx, 431.xx, 433.01, 433.11, 433.21, 433.31, 433.81, 433.91, 434.01, 434.11, 434.91, 436.xx | I63.xx |  |  |  |

Supplement 2: Terms used to search for evidence of cannabis use in the medical record

| Marijuana |
| --- |
| Marij |
| MJX (Not TMJX) |
| Marj (misspelling) |
| Cannabis |
| Cannab |
| Canab (misspelling) |

* each term should include a space before and after while searching.

Supplement 3. Research Assistant Training Process

| Training Component | Steps |
| --- | --- |
| Medical Chart Abstraction | 1. Trainee abstracts at least 2 records with full guidance of Trainer 2. Trainee abstracts ≥2 additional records with partial guidance; Trainer addresses all questions/concerns of Trainee during abstractions 3. Trainee abstracts at least 1 training batch (25 records previously abstracted by Trainer)    1. If >1 discrepancy* in first training batch, Trainee abstracts 1 additional batch    2. Repeat new training batch(es) until no discrepancies found |
| Telephone Interviews | 1. Trainee reviews staff-developed Technology Guide and Interview Guide 2. Trainer goes through interview with Trainee and introduces different probing methods 3. Trainee does simulation calls with other trained Research Assistants (Ras)    1. Trainer provides direct feedback while listening to simulation calls with Trainee in 1-on-1 session    2. Continue simulation calls until Trainee’s interview skills are well-developed 4. Trainee listens to recorded interviews of trained Ras while Trainer conducts Quality Assurance (QA); Trainer addresses all questions/concerns of Trainee    1. Continue until Trainee’s interview skills are well-developed 5. Once interview skills well-developed, Trainee does first 10 interviews with participants    1. Trainer provides feedback using QA Form (see below)    2. If average overall score on first 10 interviews is ≥95%, Trainee is considered certified to interview    3. If average score <95%, Trainee conducts another 5 interviews and Trainer provides feedback and score using QA Form    4. Repeat until average score ≥95% 6. Once certified, Trainee moves into QA phase |
| Quality Assurance (QA) | - RAs record every call - Two calls per RA randomly selected and reviewed each week using staff-developed QA Form - QA Form   - Each section scored for:     - Script compliance and answer coding     - Probing   - Overall survey scored for:     - Problem solving abilities     - Call etiquette     - Script compliance and answer coding     - Total score |

*Based upon inclusion/exclusion criteria and reasons for exclusion

Supplement 4. Source Material for Interview Tool

| **Scale** | **Source Material** | **Reference** |
| --- | --- | --- |
| Overall Health Questions | Short Form Survey (SF-36) | Ware JE Jr, Sherbourne CD. The MOS 36-item short-form health survey (SF-36). I. Conceptual framework and item selection. Med Care. 1992 Jun;30(6):473-83. PMID: 1593914.  <https://www.rand.org/health-care/surveys_tools/mos/36-item-short-form.html> |
| Mobility | Health and Retirement Study (HRS) | <https://hrs.isr.umich.edu/about> |
| Physical Activity | International Physical Activity Questionnaire (IPAQ) | <http://www.ipaq.ki.se/> |
| Alcohol Use | Alcohol Use Disorders Identification Test (AUDIT C) | Bush K, Kivlahan DR, McDonell MB, Fihn SD, Bradley KA, for the Ambulatory Care Quality Improvement Project (ACQUIP). The AUDIT Alcohol Consumption Questions (AUDIT-C): An Effective Brief Screening Test for Problem Drinking. Arch Intern Med. 1998;158(16):1789–1795.  <https://www.mdcalc.com/audit-c-alcohol-use> |
| Tobacco Use | Adapted from Psychiatric Research Interview for Substance and Mental Disorders for DSM-V (PRISM-V), National Health Interview Survey (NHIS) | PRISM-V: Hasin DS, Trautman KD, Miele GM, Samet S, Smith M, Endicott J. Psychiatric Research Interview for Substance and Mental Disorders (PRISM): reliability for substance abusers. Am J Psychiatry. 1996 Sep;153(9):1195-201. PMID: 8780425.  NHIS: <https://www.cdc.gov/nchs/nhis/index.htm> |
| Marijuana Use | Cannabis Assessment Tool (CAT-1) |  |
| Secondhand Smoke Exposure | Adapted from NHIS | <https://www.cdc.gov/nchs/nhis/index.htm> |
| Illicit Drug Use | Adapted from Coronary Artery Risk Development in Young Adults (CARDIA) | <https://www.cardia.dopm.uab.edu/exam-materials2/data-collection-forms> |
| Mood | Patient Health Questionnaire (PHQ-9) | Kroenke K, Spitzer RL, Williams JB. The PHQ-9: validity of a brief depression severity measure. *J Gen Intern Med*. 2001;16(9):606-613.  <https://www.mdcalc.com/phq-9-patient-health-questionnaire-9> |
| PTSD | PTSD Checklist-5 (PCL-5) | Blevins CA, Weathers FW, Davis MT, Witte TK, Domino JL. The Posttraumatic Stress Disorder Checklist for DSM-5 (PCL-5): Development and Initial Psychometric Evaluation. J Trauma Stress. 2015 Dec;28(6):489-98. Epub 2015 Nov 25. PMID: 26606250.  [https://www.ptsd.va.gov/professional/ assessment/documents/using-PCL5.pdf](https://www.ptsd.va.gov/professional/assessment/documents/using-PCL5.pdf) |
| Aspirin | CAT-1 |  |
| SES | Adapted from CARDIA and HRS | CARDIA: <https://www.cardia.dopm.uab.edu/>  HRS: <https://hrs.isr.umich.edu/about> |

Supplement 5. Baseline Characteristics of Individuals who Participated in the Telephone Interview Compared to Individuals who Refused to Participate or Were Not Reached

|  |  | ***non-respondents*** | ***respondents*** |
| --- | --- | --- | --- |
|  |  | mean(sd) | |
| ***Age*** |  | 67.1 (0.81) | 67.7 (1.11) |
|  |  | N (%) | |
| ***Gender*** |  |  |  |
|  | *Male* | 1941 (98.2%) | 4196 (97.9%) |
|  | *Female* | 35 (1.8%) | 89 (2%) |
| ***Race*** |  |  |  |
|  | *White* | 1481 (75%) | 3405 (79%) |
|  | *Black* | 337 (17%) | 714 (17%) |
|  | *Other* | 158 (8%) | 166 (4%) |
| ***Marital Status*** | |  |  |
|  | *Married/ Partner* | 932 (47%) | 2092 (49%) |
|  | *Other* | 1044 (53%) | 2193 (51%) |


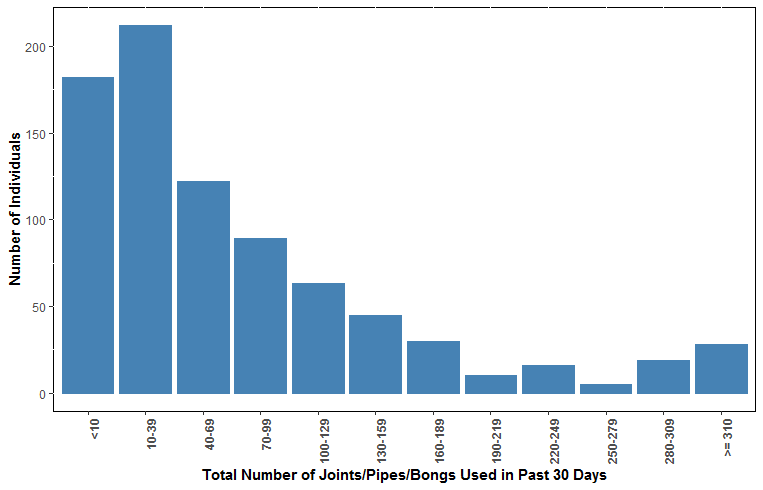


Supplement 6: Frequency of Use Per Month.
